# Letter to the Editor from Wagner et al: “An Unusually Prolonged Case of FGF23-Mediated Hypophosphatemia Secondary to Ferric Carboxymaltose Use”

**DOI:** 10.1210/jcemcr/luae078

**Published:** 2024-05-14

**Authors:** Sonja A Wagner, Benedikt Schaefer, Heinz Zoller

**Affiliations:** Christian Doppler Laboratory for Iron and Phosphate Biology, Medical University of Innsbruck, 6020 Innsbruck, Austria; Department of Internal Medicine I, Medical University of Innsbruck, 6020 Innsbruck, Austria; Department of Internal Medicine I, Medical University of Innsbruck, 6020 Innsbruck, Austria; Christian Doppler Laboratory for Iron and Phosphate Biology, Medical University of Innsbruck, 6020 Innsbruck, Austria; Department of Internal Medicine I, Medical University of Innsbruck, 6020 Innsbruck, Austria

Dear Editor,

The patient reported by Ipsa Arora and coworkers adds new insights to the increasing body of evidence that ferric carboxymaltose (FCM) can cause severe and long-lasting complications. The severity and duration of FCM-induced hypophosphatemia in this case is highlighted by the fact that the patient requires medical treatment over 3.5 years after a single treatment course with FCM. Oral phosphate was poorly tolerated, and hypophosphatemia was only temporarily corrected by intravenous phosphate, which is in line with FCM-induced hypophosphatemia being caused by renal phosphate wasting [1].

Treatment of FCM-induced hypophosphatemia represents a therapeutic challenge, which—in the present case—was successfully met with calcitriol. This is a rational approach when patients have developed secondary hyperparathyroidism as a consequence of the complex changes in mineral metabolism induced by FCM [2].

A secondary analysis of data from FCM-treated patients in the pooled PHOSPHARE IDA trials has shown that there were 52% (46/87) of FCM-treated patients who developed persistent hypophosphatemia. The mean increase in parathyroid hormone (PTH) from baseline was 50 pg/mL and severe iron deficiency, indicated by low ferritin levels, was the only significant predictor of persistent hypophosphatemia, apart from baseline phosphate [3].

The cascade that causes secondary hyperparathyroidism after FCM has been summarized as *6H-syndrome*. Fibroblast growth factor 23 (FGF23) could trigger this sequence, but—as in this case—patients may still suffer from hypophosphatemia long after FGF23 has recovered. The 6H-syndrome is an acronym for hypophosphatemia caused by high FGF23 leading to hyperphosphaturia and hypocalcitriolemia, which results in hypocalcemia and compensatory secondary hyperparathyroidism [4]. The patient presented here had hypophosphatemia and hyperphosphaturia (TmP/GFR) but intact FGF23, calcitriol, calcium, and PTH concentrations were all within the reported reference ranges.

The present case report therefore introduces the novel concept of “inappropriately normal FGF23.” The biologically active form of FGF23—intact FGF23—was found to be normal, but the exact time when it was measured after the infusion is not reported. This raises the question whether persistent hypophosphatemia after FCM was caused by “inappropriately normal FGF23” or by “inappropriately normal PTH”? Contrary to what is implied in the title, the latter was probably the rationale for calcitriol treatment, which has been effective in similar cases, despite the fact that in vitro calcitriol is a strong inducer of FGF23.

Finally, the authors discuss 2 potential causes of persistent hypophosphatemia. First, the “large cumulative dose administered in a short period”—2 doses of 750 mg of FCM 1 week apart. Considering that this exact dose and timing are in accord with the FDA drug label, this cause is unlikely. The second proposed cause, the genetic mutation in PKD1 is inferred from the finding that conditional disruption of PKD1 in osteoblasts leads to bone loss in mice. This is also unlikely because the patient had “normal bone mineral density for age.”

Taken together, FCM is associated with an unpredictable risk for severe, long-lasting, and potentially irreversible complications, which is preventable by choosing alternative iron formulations such as low-molecular iron dextran or ferric derisomaltose.

## Abbreviations

FCM: ferric carboxymaltose
FGF23: fibroblast growth factor 23
PTH: parathyroid hormone
